# Muographic mapping of the subsurface density structures in Miura, Boso and Izu peninsulas, Japan

**DOI:** 10.1038/srep08305

**Published:** 2015-02-09

**Authors:** Hiroyuki K. M. Tanaka

**Affiliations:** 1 Earthquake Research Institute, The University of Tokyo, 113-0032 Tokyo

## Abstract

While the benefits of determining the bulk density distribution of a landmass are evident, established experimental techniques reliant on gravity measurements cannot uniquely determine the underground density distribution. We address this problem by taking advantage of traffic tunnels densely distributed throughout the country. Cosmic ray muon flux is measured in the tunnels to determine the average density of each rock overburden. After analyzing the data collected from 146 observation points in Miura, South-Boso and South-Izu Peninsula, Japan as an example, we mapped out the shallow density distribution of an area of 1340 km^2^. We find a good agreement between muographically determined density distribution and geologic features as described in existing geological studies. The average shallow density distribution below each peninsula was determined with a great accuracy (less than ±0.8%). We also observed a significant reduction in density along fault lines and interpreted that as due to the presence of multiple cracks caused by mechanical stress during recurrent seismic events. We show that this new type of muography technique can be applied to estimate the terrain density and porosity distribution, thus determining more precise Bouguer reduction densities.

Conventional gravimetric analysis assumes a uniform density distribution above the reference surface, in order to derive a Bouguer gravity anomaly map. This assumed density is called the Bouguer reduction density. Uncertainties of the Bouguer reduction density affect the result when one estimates the underground density distribution. In order to reduce these uncertainties, several analysis methods to estimate terrain density distribution have been proposed^1^,^2^,^3^. However, even the most recent method proposed by Nawa et al. (1997)^3^ generates an erroneous density distribution if the target area contains steep gradient in the Bouguer anomaly and topography (e.g., the area crossing a fault). The inverse problem in gravimetry, i.e. the computation of the density function (location, form and density of the disturbing mass) from the observed gravity data leads to an integral equation for which no unique solution exists. Hence, it is not possible to directly obtain absolute density distributions using this method.

Starting with George's 1955 mine gallery experiment^4^, muography has shown that this technique can derive the absolute average density of matter along the muon path and have visualized shallow crustal density distribution inside volcanoes^5^,^6^,^7^,^8^ and fault zones^9^,^10^ as well as archaeological objects^11^ with better spatial resolution (in the order of a few m) than possible with the conventional techniques, including high resolution seismic tomography^12^,^13^. Muography can also be utilized to monitor temporal density variations inside large objects, due to, e.g., magma dynamics^14^ and underground water table changes^15^, by taking advantage of the almost constant rate of muon flux arriving on Earth. However, these measurements were all taken by placing a detector at a single location and by recording muon events there for several days (until obtaining the statistically sufficient amount). Depending on the detector's viewing angle and the distance to the target, a feasible target size is limited to approximately 1 km in diameter. Expanding upon this standard technique, we have developed a new muography technique by taking advantage of a power-effective, portable detection system and the presence of traffic tunnels densely dispersed throughout the country. The technique makes it possible for us to measure (with greater speed and accuracy) the shallow density at different locations to map out the density distribution of significantly larger areas.

Cosmic ray muons are generated in the Earth's atmosphere as the secondary cosmic rays and their differential vertical flux has been precisely measured^16^. Integrated vertical open-sky muon flux *I*_0_ is known to be ~70/m^2^▪sr▪s^17^, and is reduced after passing through matter. This reduction can be calculated by integrating the differential flux over the energy range between the critical energy (the minimum energy that the muon can penetrate the target object) and infinity. Critical energy can be precisely calculated within the framework of the standard model of particle physics^18^. For example, the vertical muon flux is reduced by a factor of 27 after passing through 100 m of water equivalent (m.w.e.). The reduction of muon flux as a function of the density length (density times matter length) has been quantitatively studied by many underground experiments and thus, there are almost no uncertainties in these theories^18^. Furthermore, most muons traverse matter in a linear trajectory^19^. This means that the path length of muons can be precisely determined by reading the outer geometry of the target volume. The open-sky muon counting rate (*N*_0_) is measured outside the tunnels. To calculate first the density length of the overburden *X* in units of m.w.e and then the average density <*ρ*>, the theoretically predicted reduction factor *I*_0_/*I* is compared with the recorded muon counting rate ratio (called the observational reduction factor) *N*_0_/*N*_μ_ between the data collected inside and outside of the tunnel along with the geometrical thickness of the target *L* (measured from, e.g., a topographic map). Since we consider the *N*_0_/*N*_μ_ ratio, we can neglect the detector's efficiency. The average density of the overburden is determined by dividing the density length of the overburden by the geometrical thickness of the overburden, namely, <*ρ*> = *X*/*L*. If the rock thickness is the same, the reduction factor depends only on the density of the material, i.e., muons traveling through low-density materials survive in greater numbers and vice versa. For example, the vertical muon flux is attenuated to 2.9/m^2^▪sr▪s and 2.1/m^2^▪sr▪s after passing through 90-m.w.e. rock and 110-m.w.e. rock respectively. Therefore, when the target density varies by ±10%, the muon reduction factor is altered by ~
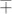
16%. The time (*t*) required for holding the following equation: 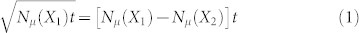
is less than 3 minutes with a 0.1-m^2^▪sr muon detector, where *N*_μ_(*X_i_*) is the muon counting rate after passing through the density length of *X_i_*. The density lengths *X*_1_ and *X*_2_ hold the following relationship: 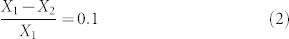
Therefore, it is in principle possible to measure the overburden rock density with this type of portable detector in a relatively short time. In this paper we report on the first muographic mapping of the shallow density distribution in three different Japanese peninsulas.

## Results

### Observations

**Figure 1:**
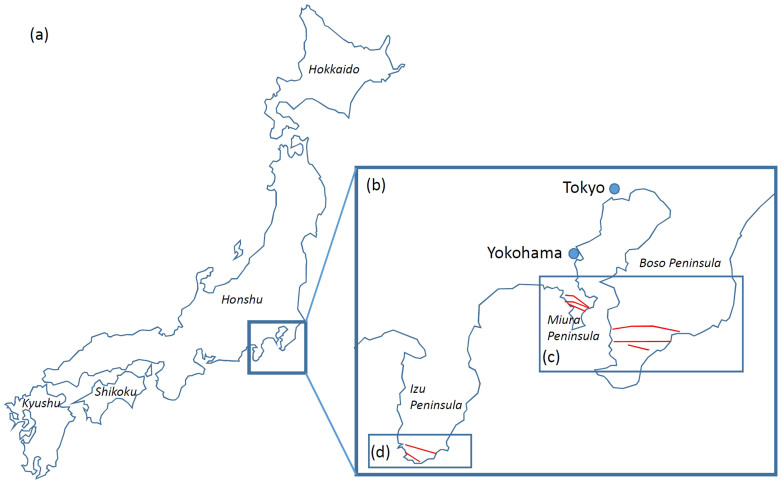
Muographically investigated areas in Japan. (a), a map showing the location of Kanto-Tokai area (indicated with the small box) of Japan (consisting of Hokkaido, Honshu, Shikoku and Kyushu Islands). Inset (b) shows the enlarged map of Kanto-Tokai area that includes cities of Tokyo and Yokohama. Insets (c) and (d) show the muographically investigated areas, i.e. Miura, South-Boso and South-Izu Peninsulas. Red lines indicate the active faults. H.K.M.T. drew the map and holds the copyright.

**Figure 2:**
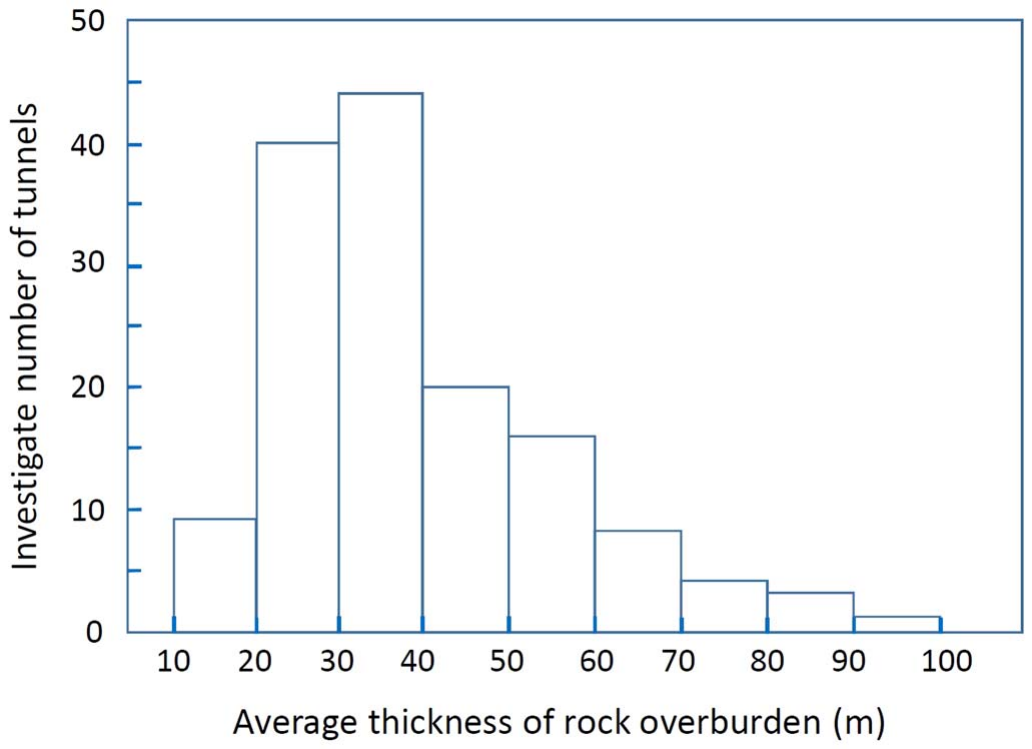
Average thickness distribution of the investigated rock overburdens. 66% of the investigated tunnels have overburdens thicker than 30 m.

We chose Miura, South-Boso and South-Izu peninsula as the areas to be imaged through with muons (Fig. 1). Miura peninsula mainly consists of an accreted sediment complex generated 15–20 million years ago on the sea floor of Pacific Ocean^20^. This complex lifted ~500 thousand years ago and eventually formed what are now Miura and Boso peninsulas. It is known that four active fault segments^21^ (Kinugasa, Kitatake, Takeyama, and Minami-Shitaura) and two active fault segments (North-Kamogawa and South-Kamogawa) cut Miura and South-Boso peninsulas, respectively^22^. Izu peninsula, on the other hand, was produced by the collision of submarine volcanoes with Honshu Island one million years ago. These submarine volcanoes were located on the Philippine Sea Plate that was drifting in the direction of Honshu Island. After this collision, these volcanoes uplifted and formed the present shape of the peninsula. Therefore, the Izu peninsula is mainly formed of volcanic rocks^20^. The sizes of the surveyed areas are 150 km^2^, 1100 km^2^, and 90 km^2^, and the number of observation points are 43, 81, and 22 for Miura, South-Boso and South-Izu peninsula, respectively. Therefore, the average size of the area per observation point is 3.5 km^2^/point (Miura), 13.6 km^2^/point (South-Boso), 4.1 km^2^/point (South-Izu). However, there is a different tunnel distribution in the various sites. The thickness of the surveyed tunnel overburdens ranges from 15 m to 100 m (Fig. 2), depending on the tunnels and the observation altitude is near sea level.

**Figure 3:**
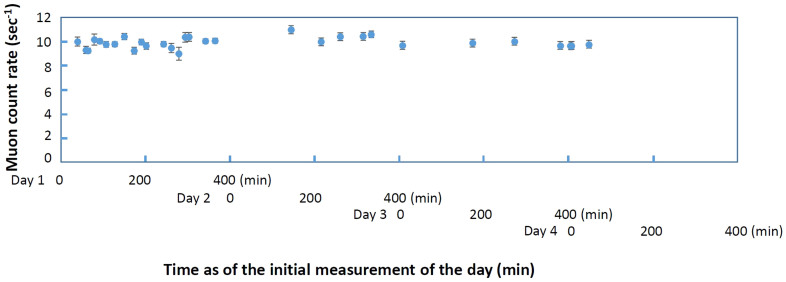
Muon counting rate as a function of time. The counting rate is plotted for different measurement dates (from Day 1 to Day 4) together with error bars (1σ). Vertical dashed lines separate the measurement dates.

The detection system we used consists of a four-fold Cockcroft-Walton type scintillation detector^23^, a Muon Readout Module^24^, a DC power supply, a lithium-ion battery (20 kAh), and a laptop computer (Apple Macbook Air) for data collection. With the exception of the computer, the total power consumption for the system was less than 8 W, so the detector could be operated more than 8 hours with one battery. Detector planes faced the zenith so that vertical muons could be collected. The active area of the detector was 0.16 m^2^sr, and the open-sky muon counting rate *N*_0_ was 10.6 muons/sec. Open sky measurements were performed between the surveyed tunnels. We assume that this flux is the same as that above the tunnels because the altitude-dependence of the muon flux is negligible in the present case (the difference in the muon flux is less than 1% between sea level and 500 m above sea level^17^. The typical time duration to move from one tunnel to next tunnel was 30 minutes, therefore the statistic error was typically less than 0.5%. Time variation in the open sky muon counting rate was 3% and did not depend on the location (Fig. 3). This number is consistent with the theoretical prediction. Tunnel overburden topography data were obtained by referring to a 1:25000 topographic map distributed by Geographical Survey Institute (GSI), Japan. GSI reported that the error in the contour height of the map is no more than ±2.5 m. This could potentially lead to a density determination error of up to ±10% and ±5% for an overburden thickness of 25 m and 50 m. However, it is unlikely that all of the contours of the overburden are systematically deviated by 2.5 m, and furthermore, the comparison between a 1:25000 topographic map and the airborne laser scanning results^25^ shows that the difference between them tends to be less than 1 m. Therefore, we assumed that the majority of contour height errors were expected to be much smaller than ±2.5 m. The measurements were taken while the vehicle was moving and the data were given by integration of the attenuated muon flux over the traveled distance. Using a global positioning system (GPS), the position of the vehicle at the entrance of the tunnel was accurately identified and the acceleration of the vehicle inside a tunnel was measured with a gyro-sensor. The positioning error inside the tunnel was typically less than a few meters that is higher than the accuracy of the digital elevation map (DEM). A more detailed description of our analytical method will be given in the Method section.

**Figure 4:**
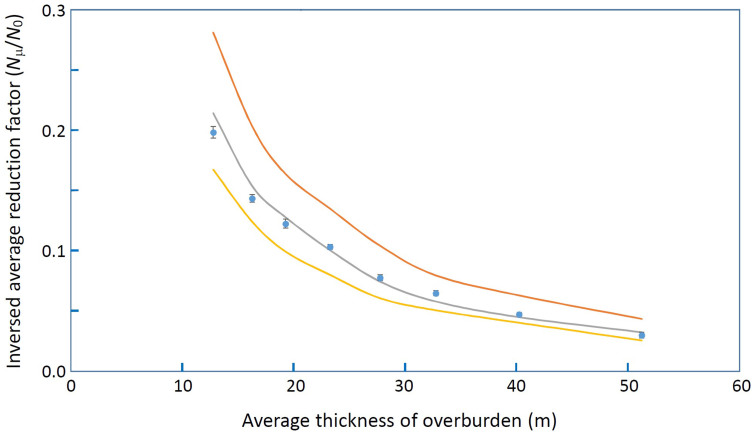
Inversed reduction factor as a function of the average thickness of the tunnel overburden. The error bars associated with the data points (blue dots) show the 1σ upper and lower limits. Theoretical curves were calculated by assuming uniform overburden densities of 2.0 g/cm^3^ (red), 2.5 g/cm^3^ (gray), and 3.0 g/cm^3^ (yellow).

Fig. 3 plots the inverse of the observational reduction factor (*N*_μ _/*N*_0_) as a function of averaged overburden thickness. The data collected from all of three peninsulas were used to plot this graph along with theoretically predicted reduction factors for different assumed densities (see Method section). By comparing the data points with these theoretical predictions, the density averaged over these three regions was deduced to be 2.51 ± 0.02 g/cm^3^. This muographically deduced density was compared with independent results from gravity studies. Nawa et al. (1997)^3^ reported the average density of tertiary sedimentary and volcanic rocks above sea level in Southwest Japan to be 2.54 ± 0.15 g/cm^3^ and 2.53 ± 0.13 g/cm^3^, respectively. These values are in agreement with our results. As shown in Fig. 4, one data point (between 30 and 40 m) shows density (2.35 g/cm^3^) much lower than 2.51 g/cm^3^. This is because the average density was affected by several low density points measured along the active fault lines as described more in detail in the following subsections and the discussion section. Since our detector is moving inside the tunnel, and cannot distinguish muons with different angles of incidence, the thickness in Fig. 4 is presented as “the average reduction factor”, which is the reduction factor calculated for a mixture of various rock thicknesses. Thus, the average reduction factor tends to be systematically lower than the reduction factor associated to the same rock thickness.

**Figure 5:**
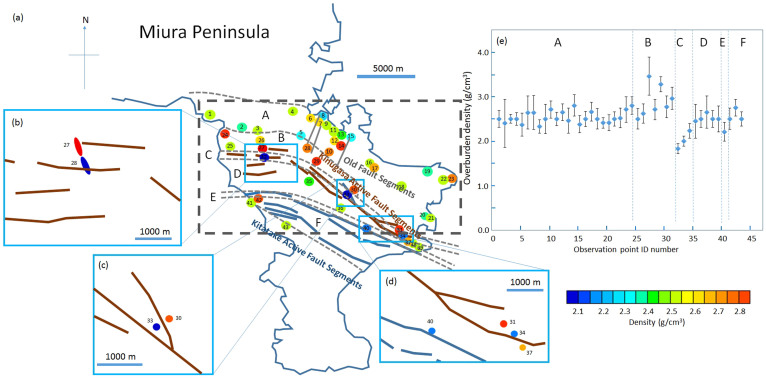
Muographic density mapping of Miura Peninsula. (a), location of the observation points in Miura Peninsula. The observation points are grouped according to the different geological features: A, middle or late Miocene marine or non-marine sediments (formed between 15 and 7 million years ago); B, late Eocene or early Miocene accretionary complex (formed between 40 and 22 million years ago); C, Kinugasa active fault segment; D, middle or late Miocene marine or non-marine sediments (formed between 15 and 7 million years ago); and E, Kitatake active fault segment; F, late Eocene or early Miocene accretionary complex (formed between 40 and 22 million years ago). Gray dashed lines indicate geological boundaries, and blue bold lines are active fault lines (the data were taken from The AIST (National Institute of Advanced Industrial Science and Technologies) Geological Map). The numbers indicate the observation point ID number (#1-#43). Actual point resolution of the measurements (that ranges between 20–100 m) is much smaller than the size of the circles on the map. Density observed at each observation point is shown with different colors ranging from low-density (blue) to high density (red). The enlarged maps show detailed views of the observation points (b) #27 and #28, (c) #30 and #33, and (d) #31, #34, #37 and #40. The plot of observed densities with error bars (1σ) as a function of the observation point ID number is shown in (e). Capital letters shown in this plot correspond to the Miura Peninsula map (a). H.K.M.T. drew the map and holds the copyright. The dotted lines show the region used for plotting Fig. 10.

**Figure 6:**
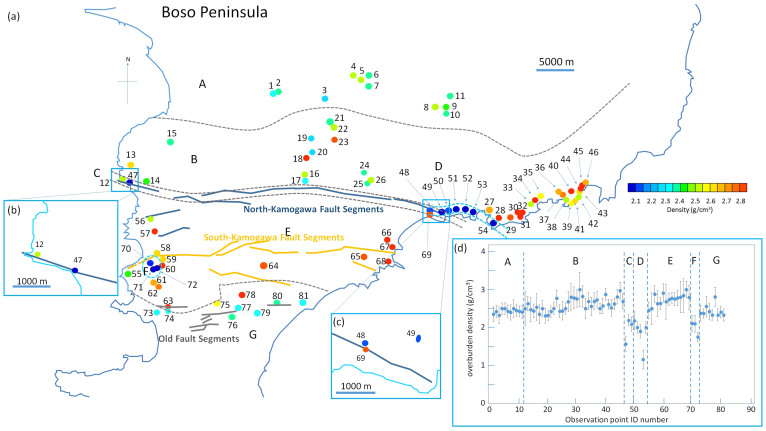
Muographic density mapping of South-Boso Peninsula. (a), location of the observation points in South-Boso Peninsula. The observation points are grouped according to the different geological features. A: early Pleistocene marine or non-marine sediments (formed between 1.7 and 0.7 million years ago); B: Miocene or Pliocene marine or non-marine sediments (formed between 22 and 1.7 million years ago); C: North-Kamogawa active fault segment; D:low-density region extending eastward towards the east of North-Kamogawa fault segment (surrounded by sky blue dashed lines); E: late Eocene or early Miocene accretionary complex (formed between 40 and 22 million years ago); F: low-density cluster found in Region E (surrounded by sky blue dashed lines); G: late Miocene or Pliocene marine or non-marine sediments (formed between 7 and 1.7 million years ago). Gray dashed lines indicate geological boundaries, and blue, orange and gray bold lines represent respectively the North-Kamogawa, South-Kamogawa active fault lines, and old (less active) fault lines (the data were taken from The AIST (National Institute of Advanced Industrial Science and Technologies) Geological Map). The numbers indicate the observation point ID number (#1-#81). Actual point resolution of the measurements (that ranges between 20–100 m) is much smaller than the size of the circles on the map. Density observed at each observation point is shown with different colors ranging from low-density (blue) to high density (red). The enlarged maps show detailed views of the observation points (b) #12 and #47 and (c) #48, #49 and #60. The plot of observed densities with error bars (1σ), as a function of the observation point ID number is shown in (d). Capital letters shown in this plot correspond to the South-Boso Peninsula map (a). H.K.M.T. drew the map and holds the copyright.

**Figure 7:**
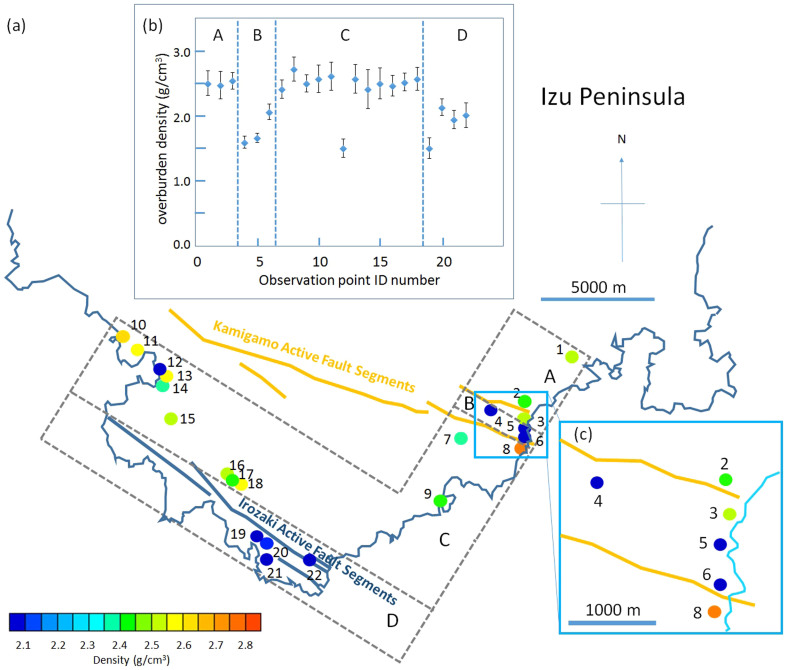
Muographic density mapping of South-Izu Peninsula. (a), location of observation points in South-Izu Peninsula. The observation points are grouped according to the different geological features: A: region north of Kamigamo active fault segment; B: Kamigamo active fault segment; C: region between Kamigamo and Irozaki active fault segements; D: Irozaki active fault segment. Gray dashed lines indicate boundaries of these four regions. The numbers indicate the observation point ID number (#1-#22). Actual point resolution of the measurements (that ranges between 20–100 m) is much smaller than the size of the circles on the map. Density observed at each observation point is shown with different colors ranging from low-density (blue) to high density (red). The plot of observed densities with error bars (1σ), as a function of the observation point ID number is shown in (b). Capital letters shown in this plot correspond to the South-Izu Peninsula map (a). The enlarged map (c) shows detailed views of the observation points from #2 to #6 and #8 (c). H.K.M.T. drew the map and holds the copyright.

**Figure 8:**
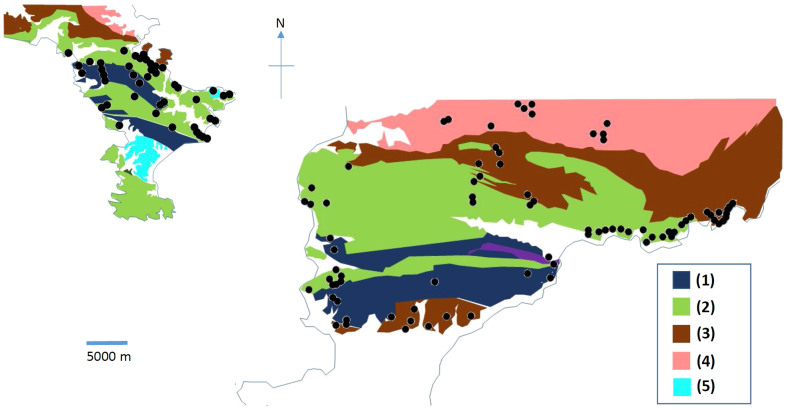
Geological maps of the Miura and Boso peninsulas. This map shows the distribution of the accretionary complex formed between 40 and 20 million years ago (1), marine and non-marine sediments formed between 15 and 7 million years ago (2), those formed between 7 and 1.7 million years ago (3), those formed between 1.7 and 0.7 million years ago (4), and those formed between 0.7 and 0.15 million years ago (5). Black dots show the locations of observation points. H.K.M.T. drew the map and holds the copyright.

Figs. 5, 6 and 7 show muon-deduced maps of above-tunnels density distributions in Miura, South-Boso and South-Izu peninsulas, respectively. These plots are overlaid with indications of active faults^21^,^22^,^26^, geological boundary lines^20^ (Fig. 8), and old fault lines (considered nearly or completely inactive) drawn for reference. Insets of each figure show plots of derived density with 1σ error bars as a function of the tunnel identification (ID) number and the magnified maps for a greated detail. Specific results from each peninsula will be more extensively discussed in the following subsections.

### Miura Peninsula

In Miura Peninsula we selected 43 observation points. Since the surveyed area was 150 km^2^, the average area per observation point was 3.5 km^2^/point. The average density of all 43 points was 2.55 ± 0.03 g/cm^3^. We divided the survey area on the map into 6 regions (from A to F) based on the geological boundaries. A: middle or late Miocene marine or non-marine sediments, B: late Eocene or early Miocene accretionary complex, C: fault zone (Kinugasa fault segment), D: middle or late Miocene marine or non-marine sediments, E, fault zone (Kitatake fault segment), and F: late Eocene or early Miocene accretionary complex. As reported in Fig. 5, contiguous and adjacent points located in the vicinity (within a 200 m) of a fault line show significantly lower density than the other points (see inset of Fig. 5). This trend can also be seen in the other peninsulas (Figs. 5 and 6). In particular, three low-density points (ID#32 (*ρ* = 1.83 ± 0.11 g/cm^3^), #33 (*ρ* = 2.00 ± 0.11 g/cm^3^), and #34 (*ρ* = 2.23 ± 0.16 g/cm^3^)) along the Kinugasa fault segment are adjacent (within 200 m) to high-density points (ID#27 (*ρ* = 3.46 ± 0.43 g/cm^3^), #30 (*ρ* = 2.77 ± 0.24 g/cm^3^), #31 (*ρ* = 2.96 ± 0.25 g/cm^3^)). This is an indication that there is a steep density gradient in this small region. Relatively fewer density points (ID#8 and #15) are distributed along the old fault lines; however they are not statistically significant. It can be also noticed that the average density of the region where the accretionary complex appears on the surface has slightly higher density than that of middle or late Miocene sediments. The average density excluding the Kinugasa and Kitatake fault segment regions is 2.60 ± 0.03 g/cm^3^.

### South-Boso Peninsula

For the region of South-Boso Peninsula, we took data from 81 observation points. The total survey area is 1100 km^2^ implying that the average area per observation point is 13.6 km^2^/point. The average density of all the 81 points is 2.50 ± 0.02 g/cm^3^. We focused particularly on the two prominent fault segments existing in South-Boso Peninsula that divide the land roughly in an East to West direction. We divided South-Boso Peninsula into seven regions (from A to G). A: the early Pleistocene marine or non-marine sediments, B: the Miocene or Pliocene marine or non-marine sediments, C: a fault zone (North-Kamogawa fault segment), D: a low density region extended towards the east of North-kamogawa fault, E: the late Eocene or early Miocene accretionary complex, F: a low density cluster found in Region E, and G: the late Miocene or Pliocene marine or non-marine sediments. Similarily to what was previously obtained for Miura Peninsula, the points showing significant lower density than the other points were located along or nearby the fault segment (in this case, North-Kamogawa fault segment). As shown in Fig. 6 (Regions D and F surrounded by sky blue dashed lines), this survey revealed two more low-density regions, one being the east extension of North-Kamogawa fault segment and the other being located near the west end of South-Kamogawa fault segment (orange bold lines in Fig. 5). There is only a small difference between the average densities of Regions B and E (2.61 ± 0.03 g/cm^3^ and 2.77 ± 0.06 g/cm^3^ respectively), while there is a large difference between Regions A and E (2.43 ± 0.05 g/cm^3^ and 2.77 ± 0.06 g/cm^3^, respectively). The average density in Region G (2.44 ± 0.06 g/cm^3^) is less than that in Region B (2.61 ± 0.03 g/cm^3^) although geology in these regions is the same. This might be related to the fact that Region G includes more old faults than Region B. The average density excluding the North- and South-Kamogawa fault segment regions was 2.59 ± 0.02 g/cm^3^, the same value as the average density of Miura Peninsula.

### South-Izu Peninsula

South-Izu Peninsula was investigated with 22 observation points and the average area per observation point was 4 km^2^/point since the surveyed area was 90 km^2^. The average density of these 22 points was 2.26 ± 0.03 g/cm^3^. Although there are two active fault segments (Irozaki and Kamigamo fault segments) extending from northwest to southeast, South-Izu Peninsula geological domains are distributed in a more random manner in comparison to Miura and South-Boso Peninsulas. Indeed, South-Izu Peninsula was generated by recurrent volcanic activities that later resulted in patches of various types of volcanic rocks (basaltic, andesitic, dacitic and rhyolitic rocks aging from middle Miocene to Pliocene) and sedimentary rocks (middle Miocene and Pliocene marine sediments). Therefore in South-Izu Peninsula, we divided the surveyed area into 4 regions (from A to D) with lines extending from NW to SE and parallel to the Irozaki fault line. Regions A and C do not include the fault, but B and D each have one fault (Irozaki and Kamigamo fault segments, respectively). As noted before, low-density points are concentrated in the regions that include fault segments. However, there is also one low-density point in Region B with a statistical significance. The average density excluding these low-density points is 2.52 ± 0.05 g/cm^3^.

## Discussion

### Comparison with geological map

**Figure 9:**
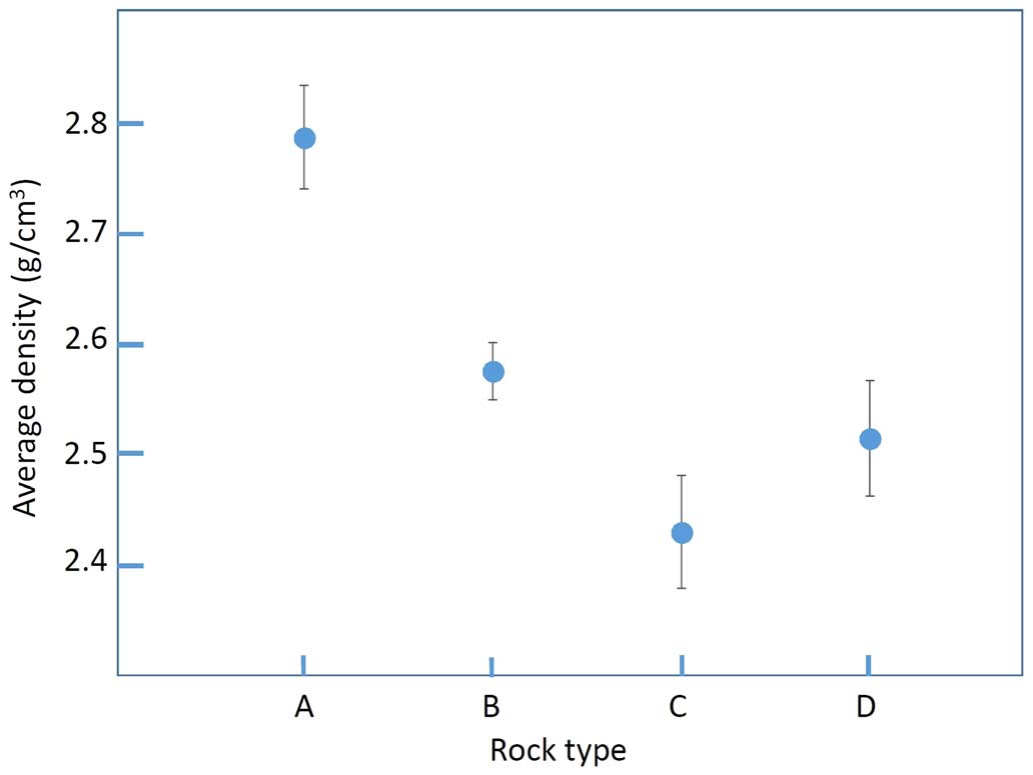
Average density determined for different rock types. Capital letters indicate rock types: A: accretionary complex formed between 40 and 22 million years ago; B: marine and non-marine sediments formed between 15 and 1.7 million years ago; C: marine and non-marine sediments formed between 1.7 and 0.7 million years ago; D: basaltic and andesitic rock formed between 7 and 1.7 million years ago.

Fig. 9 compares the average densities determined by using data points in different geological domains as indicated with dashed lines in Figs. 4, 5, and 6: (A) accretionary complex formed between 40 and 20 million years ago (2.79 ± 0.05 g/cm^3^); (B) marine and non-marine sediments formed between 15 and 1.7 million years ago (2.57 ± 0.03 g/cm^3^); (C) marine and non-marine sediments formed between 1.7 and 0.7 million years ago (2.43 ± 0.05 g/cm^3^); and (D) basaltic and andesitic rock formed between 7 and 1.7 million years ago (2.51 ± 0.05 g/cm^3^). The data collected from different regions with identical rock types were merged and averaged to attain better statistics. We can see that as a general trend, the density tends to be higher for older rock, and these values are consistent with gravimetrically determined density values reported by Nawa et al. (1997)^3^: 2.63 ± 0.09 g/cm^3^, 2.54 ± 0.15 g/cm^3^, and 2.58 ± 0.2 g/cm^3^ respectively for Mesozoic, Tertiary, and Quaternary sedimentary rocks and 2.53 ± 0.15 g/cm^3^ for Tertiary volcanic rock. As described in the following subsections, the data points located on the fault line were excluded in this plot. Overall, higher density rocks are detected in older geological domains. A closer inspection of Figure 7 reveals an interesting density gap between tertiary and quaternary sediments. Volcanic rocks seem to have almost the same density as sedimentary rocks of the same age.

### Comparison with an active fault map

One common feature of the muographic maps of Miura, South-Boso and South-Izu peninsulas is the low bulk density along active fault lines. The fact that the points are not precisely on the fault linesis explained by the error on the location of the fault lines shown Figs. 5, 6 and 7, amounting toa few hundred meters. In a previous experiment, Tanaka et al. (2011)^9^ used muons to image a fault zone along Itoigawa-Shizuoka Tectonic Line (ISTL) and observed changes due to the rainwater permeation through cracks in the rock. The same data were later re-analyzed by Tanaka and Muraoka (2013)^10^ who observed a density decrease towards the fault plane (1.0-1.5 g/cm^3^ within about 10 m from the fault plane) while higher densities (1.5–2.5 g/cm^3^) were measured farer away from this point (~20 m from the fault plane). Based on these results, we interpreted that these low-density points indicate a higher rate of fracturing, in turn resulting from past seismic events. Three low-density points observed in Miura Peninsula were adjacent to high-density points. This indicates a steep density gradient around the fault plane. Although the fault activity levels are different between Miura Peninsula and ISTL, the phenomenon of density recovery occurring within 200 m of the fault plane was consistent with the distance as observed in ISTL, and therefore we interpreted that this density pattern is typical of fault zones. Furthermore, the adjacent low-density points measured along the fault line show relatively higher density than average. Since higher density rocks are likely to be mechanically stronger, such rocks might have terminated the fault developments. Two low-density points located near the west end of South-Kamogawa fault (Fig. 6) and one low-density point in Region B of South-Izu peninsula show a similar pattern, and thus we made a hypothesis that they are also located on an active fault plane that has not yet been included in the active fault database of Japan.

**Figure 10:**
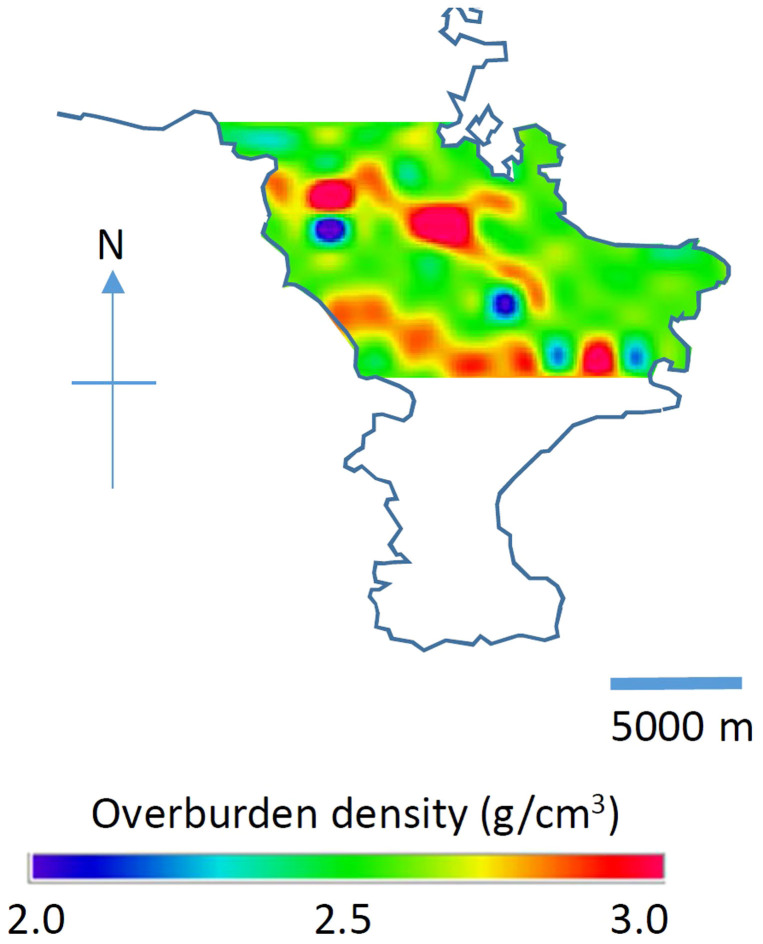
Muographic image of Miura peninsula. The colors in this map indicate the terrain density. The region used for plotting this figure is shown by the dotted lines in Fig. 5. H.K.M.T. drew the map and holds the copyright.

Another interesting feature we can see on the maps are relatively low-density points on non-active fault lines as shown in Figs. 5 (#8 and #15) and 6 (#73, #74, #77, #79, and #81). Although the deviations of each point are not statistically significant, as a whole the points are systematically lower than the other regions. For example, the average density (2.37 ± 0.06 g/cm^3^) of Region G in Fig. 6 (excluding the data points #78 and #75 clearly unrelated to the faults) deviates by more than 3σ from the average density (2.61 ± 0.03 g/cm^3^) of Region B in Fig. 6. The statistical accuracy is not strong enough to be conclusive, however we surmise that the bulk density along the fault lines may correspond to activity levels of the faults since these low-density regions may indicate rock with internal cracks susceptible to frequent fault activities. In Fig. 10, a muographic subsurface image of Miuira Peninsula is shown for the purpose of visualization. This image was produced by dividing the landmass into a 1 × 1 km^2^ grid and if more than one observation points existed in each grid cell, the average density was derived. If an observation point does not exist in a grid square, the density of the grid was represented as having the average density determined in each geological domain as defined in Figs. 4 and 5. In Fig. 10 the areas where the most grid cells have no observation points (southeastern part of Region F) are excluded. The region used for plotting Fig. 10 is shown by the dotted line in Fig. 5. The generated matrix was then interpolated with a spline function so that the grid size became 0.1 × 0.1 km^2^.

### Comparison with gravimetric survey

Nawa et al. (1997)^3^ derived density distribution above sea level in Southwest Japan. Although their survey only covers South-Izu peninsula and the point resolution is limited (12 × 12 km^2^), the results can be directly compared with the muographic results. Results of the gravimetric survey showed that the density of South-Izu peninsula ranges between 2.0 and 2.3 g/cm^3^. This value was not consistent with their laboratory density measurement results for tertiary volcanic rocks (2.51 ± 0.19 g/cm^3^). Muographically derived average density of all the 22 observation points in South-Izu peninsula is 2.26 ± 0.03 g/cm^3^, a value that is in agreement with the gravity results of Nawa et al. (1997)^3^. However, if the observation points in the fault regions are disregarded, the average density increases to 2.52 ± 0.05 g/cm^3^, a value which is consistent with the laboratory data published by the same authors. We can conclude that the rock density deduced from gravity data is lower because of the highly fractured (porous) rocks along active faults.

### Method limitations

The present muography technique utilizes traffic tunnels. Accurate geometric tunnel information (height and width) can be obtained, and its errors are negligible for the purposes of the measurement. Therefore, only the external geometry of the rock overburden and the number of muon counts determine the density measurement accuracy. Larger number of muon counts are in principle recorded when we use a larger detector or prolong the measurement duration. In order to obtain the geometry of a rock overburden, a precise topography map can be used. For this purpose, we utilized the 1:25000 topographic map freely distributed by Geographical Survey Institute (GSI), Japan, that features a vertical accuracy of ±2.5 m. Recently, an airborne laser scanning method has been developed for mapping precise topography^25^. This technique can improve the accuracy of the topography measurements (the error level is less than 1 m) and will drastically improve the accuracy in muographic measurements in the future.

Another limitation is that the distribution of the observation points depends on the geometry of the tunnel network. However, under certain conditions, complementary measurements could be taken from the ground, exploiting near-horizontal muons, to image a target to the side of the detector. Since the near horizontal muon flux is 10-100 times less than vertical flux and the horizontal paths through matter tends to be much longer than vertical paths, a larger detector will be necessary. However, we anticipate that data acquired using this technique may complement the data described in this paper.

With the objective of demonstrating this new muography technique, we mapped the shallow density distribution in Miura, South-Boso and South-Izu peninsulas. Our proposed method is capable of differentiating densities located above sea level from that below sea level only when the altitude of most of the observation points are near sea level. However, this upgraded muography technique has been shown to have potential to estimate more accurately the Bouguer reduction density. Moreover, the resulting muographic density maps from the three studied areas always show strong density decreases along the active fault lines. This density reduction is interpreted as being due to the higher degree of fracturing along the fault plane. We also observed relatively low density along the old (or less active) fault lines in South-Boso peninsula. Local bulk rock density might be related to the degree of fault activity. Lately, improved seismic source location methods have been proposed^27^,^28^,^29^,^30^. This technique in conjunction with muography can possibly improve in positioning the seismic faults. Further investigations and case studies are necessary to confirm or rule out this possibility.

The underground imaging technique proposed in the present study can be applied to subsurface geological studies, hydrological monitoring, and natural resource exploration. It also permits to retrieve a more precise Bouguer reduction density for gravimetric surveys.

## Methods

### Apparatus

Fig. 9 shows the schematic view of our portable muography detection system along with a block diagram describing the signal processing procedure. The main component of our system is the muon detector, consisting of four sheets of plastic scintillator (ELJEN EJ-200) connected to Hamamatsu H7724-based Cockcroft-Walton photomultiplier tubes (CW-PMTs)^23^ via an acryl light pipe. The dimension of each scintillator sheet is 40 × 30 × 2 cm^3^, corresponding to a maximum active area of 1200 cm^2^. The four detection planes are at a reciprocal distance of 8 cm, implying an effective active area of the detector of 0.16 m^2^ sr. This area is obtained by multiplying the active area (0.12 m^2^) by the entire solid angle spanned by the detector (1.3 sr). The output signal from the four PMTs is fed into the Muon Readout Module [Uchida et al. 2010], where analog pulses from PMTs are converted to digital signals to be sent to a Field Programmable Gate Array (FPGA) for logical processing. A datum is recorded only if the four PMTs output a signal at the same time (within 100 ns). Data are stored in the FPGA flash memory in the form of a hypertext makeup language (HTML) file. The HTML file is also updated when all four PMTs output the signal. The personal computer (Apple MacBook Air) displays the HTML files in real-time, through the web browser. Event arrival time is determined by utilizing the internal clock of the PC; the time error is ±0.5 s, implying an error of ±0.5% in the measuring muon flux, that is negligible in this work. Power was supplied by a 20-kAh lithium ion battery and power consumption of the entire system was less than 10 W including voltage regulators. The detector mostly collected vertical muons since the active area faced the zenith. About 80% of muons arrived within the zenith angle region ±30°. 

### Measurements

**Figure 11:**
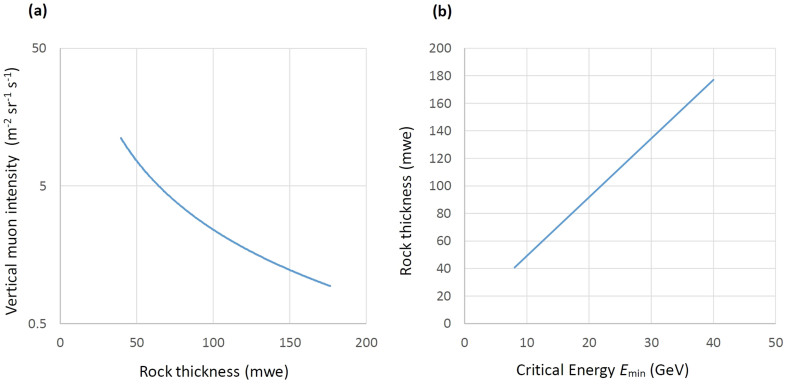
Vertical muon flux after passing through rock. Vertical flux as a function of rock thickness (in units of meter water equivalent (m.w.e.)) (a) was derived by integrating the differential vertical flux over the range between the minimum energy (*E*_min_) and infinity. The minimum energy that the muon can penetrate the target rock with a given thickness is shown in (b).

**Figure 12:**
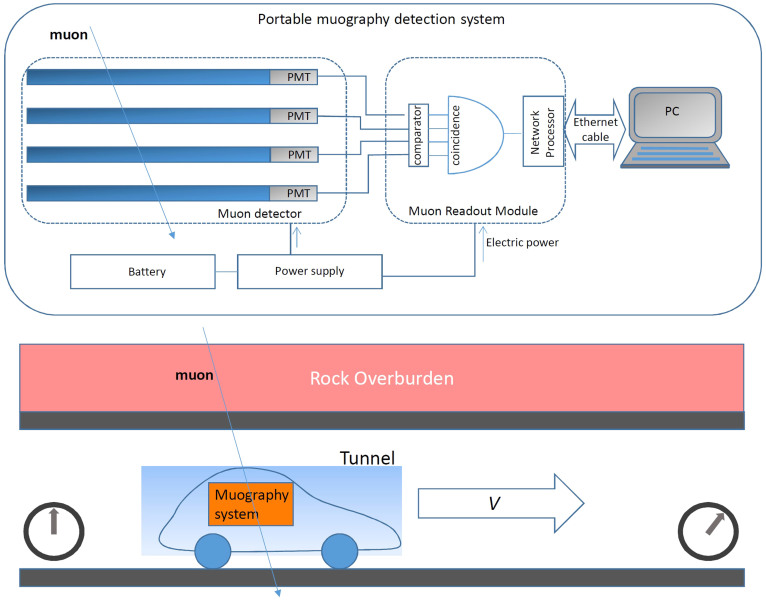
Portable muography detection system and measurement principle. Coincidence signals emitted from the four–fold muon detector are processed by the Muon Readout Module and read by a personal computer in real-time (upper). A muography detection system moving inside a tunnel with a uniform velocity *V* records muon events that cross the rock overburden. Clocks represent the beginning and ending time of the measurement.

Although vertical muon flux has been measured by several groups, there are large systematic discrepancy among the measurements, thus we utilized the vertical muon flux reported by the BESS Collaboration^16^. The muon flux was measured in Tsukuba, Japan, which is located near our observation sites, and the integrated open-air vertical flux is 68 m^−2^sr^−1^s^−1^. Since the BESS detector's angular acceptance is ±12°, we extrapolated the flux to apply the BESS flux to our measurement according to the law *I* ∝ cos^2^(*θ*), where *θ* is the arriving angle from zenith. The error in the integrated flux due to this extrapolation process was estimated to be less than 1%. Fig. 11 shows the vertical muon intensity after passing through rock with a given thickness. The total number of muon counts (*N*_μ_) recorded by the detector moving linearly along the *z*-axis inside a tunnel with a uniform velocity *V* is given by the following equation: 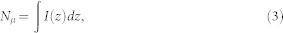
where 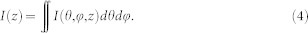
*I* (*θ, ϕ, z*) is the muon intensity after passing through the rock overburden and where its thickness *X* is a function of zenith (*θ*), azimuth (*ϕ*) angles and the location (*z*) inside the tunnel. Here, since the velocity of the detector is uniform, *z* is a linear function of *t*: 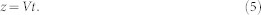
Since the critical energy (*E*_c_) can be calculated once the thickness *X* ( = *ρ* × *L*) is given^17^, *I* (*θ, ϕ, z*) is derived by integrating the vertical muon flux^16^ over the energy region between *E*_c_ and infinity. *X* (*θ, ϕ, z*) is derived by referring to the 1:25000 topographic map distributed by Geographical Survey Institute (GSI), Japan. Since *X* (*θ, ϕ, z*) varies over the distance inside the tunnel, *I* (*z*) is integrated over the distance inside the tunnel as shown in Eq. (3). If the tunnel is not linear, Eq. (3) is calculated along the tunnel. A differential vertical muon flux is assumed for the spectrum shape, but the intensity is assumed to be proportional to cos^2^(*θ*). Our muography detection system was installed in a car, and moved inside the tunnel with a uniform velocity as shown in the lower panel of Fig. 12. The data collected at the both ends of the tunnel (typically 20–30 m from the end) was discarded in order to remove the erroneous data from overburdens that were not sufficiently thick. The beginning and ending times of the measurement were recorded, and Eq. (3) was integrated over the measurement time range for a given *V* and various density values in order to compare with the muon counts recorded inside the tunnel.

The distance over which a single measurement was performed inside the tunnel is up to 500 m. Since this distance is much smaller than the scale of the area (>10 × 10 km^2^), we defined a region where data have been collected as an observation point.

## Author Contributions

H.K.M.T. wrote the text, figures, and reviewed the manuscript.
